# Identification of Key Modules and Hub Genes of Annulus Fibrosus in Intervertebral Disc Degeneration

**DOI:** 10.3389/fgene.2020.596174

**Published:** 2021-01-27

**Authors:** Hantao Wang, Wenhui Liu, Bo Yu, Xiaosheng Yu, Bin Chen

**Affiliations:** ^1^Department of Spine Surgery, School of Medicine, Renji Hospital, Shanghai Jiao Tong University, Shanghai, China; ^2^Department of Orthopedics, School of Medicine, Renji Hospital, Shanghai Jiao Tong University, Shanghai, China; ^3^Plastic & Reconstructive Surgery of the First Affiliated Hospital of Zhengzhou University, Zhengzhou, China; ^4^Department of Medicine, Lincoln Medical Center, Bronx, NY, United States

**Keywords:** intervertebral disc degeneration (IDD), weighted gene co-expression network, gene ontology, therapeutic target, annulus fibrosus

## Abstract

**Background:** Intervertebral disc degeneration impairs the quality of patients lives. Even though there has been development of many therapeutic strategies, most of them remain unsatisfactory due to the limited understanding of the mechanisms that underlie the intervertebral disc degeneration.

**Questions/purposes:** This study is meant to identify the key modules and hub genes related to the annulus fibrosus in intervertebral disc degeneration (IDD) through: (1) constructing a weighted gene co-expression network; (2) identifying key modules and hub genes; (3) verifying the relationships of key modules and hub genes with IDD; and (4) confirming the expression pattern of hub genes in clinical samples.

**Methods:** The Gene Expression Omnibus provided 24 sets of annulus fibrosus microarray data. Differentially expressed genes between the annulus fibrosus of degenerative and non-degenerative intervertebral disc samples have gone through the Gene Ontology (GO) and pathway analysis. The construction of a gene network and classification of genes into different modules were conducted through performing Weighted Gene Co-expression Network Analysis. The identification of modules and hub genes that were most related to intervertebral disc degeneration was proceeded. In order to verify the relationships of the module and hub genes with intervertebral disc degeneration, Ingenuity Pathway Analysis was operated. Clinical samples were adopted to help verify the hub gene expression profile.

**Results:** One thousand one hundred ninety differentially expressed genes were identified. Terms and pathways associated with intervertebral disc degeneration were presented by GO and pathway analysis. The construction of a Weighted Gene Coexpression Network was completed and clustering differentially expressed genes into four modules was also achieved. The module with the lowest *P*-value and the highest absolute correlation coefficient was selected and its relationship with intervertebral disc degeneration was confirmed by Ingenuity Pathway Analysis. The identification of hub genes and the confirmation of their expression profile were also realized.

**Conclusions:** This study generated a comprehensive overview of the gene networks underlying annulus fibrosus in intervertebral disc degeneration.

**Clinical Relevance:** Modules and hub genes identified in this study are highly associated with intervertebral disc degeneration, and may serve as potential therapeutic targets for intervertebral disc degeneration.

## Introduction

Low back pain (LBP), one of the most common musculoskeletal diseases, is estimated that up to 84% of the population suffer from LBP at least once in their life (Walker, 2000; Shen et al., 2018). Intervertebral disc degeneration (IDD), resulting from degenerative and inflammatory changes, promote neurovascular ingrowth into the disc and accounts for between 26 and 42% of LBP (Luoma et al., 2000; Kadow et al., 2015). Current approaches of the treatment of IDD include conservative therapies such as physiotherapy, anti-inflammatory medication, and surgical interventions including spinal fusion and disc arthroplasty. However, the clinical results of these treatments are suboptimal and a comprehensive understanding of the biological causes of IDD is required to develop improved therapies (Rao and Cao, 2014; Kadow et al., 2015).

Several studies have been conducted using microarray to investigate biomarkers and key pathways in IDD. This information not only enhances our understanding of IDD, but also highlights potential therapeutic targets. The Wnt pathway was found to be downregulated in early IDD by Smolders et al. (2013). Gruber et al. identified differentially expressed genes associated with pain, nerves and neurotrophin, and mitochondrial dysfunction, while several aberrantly expressed long non-coding RNAs (lncRNAs) were identified by Gruber et al. (2011, 2012), and Wan et al. (2014). Despite important advances in the clarification of the potential pathogenesis of IDD are achieved using high throughput microarray analysis, this established method has failed to generate a comprehensive overview of the gene network of IDD. A common practice in microarray data analysis is to apply a double filter to differentially expressed genes (DEGs) based on fold changes in expression and *t*-test *P*-values in comparisons between different groups (Zhang and Cao, 2009). However, lists of DEGs fail to elucidate the interactions among genes (Wu et al., 2013). Furthermore, downstream genes usually have greater variance resulting in their higher ranking than upstream genes, which is the key driver of disease (Naylor et al., 2010).

A number of co-expression network algorithms have been developed to investigate interactions among genes, including Weighted Gene Co-expression Network Analysis (WGCNA) (Serin et al., 2016). This algorithm is broadly applied in various fields, including lncRNA profiling of IDD (Langfelder and Horvath, 2008; Chen et al., 2015). WGCNA can be applied to high-throughput microarray or RNA-seq data sets to find clusters (modules) of highly correlated genes, using modular intrinsic genes or in-model central genes to pair these clusters Summarize, correlate modules with each other and with external sample traits, and use them to calculate module membership metrics (Pei et al., 2017). This method has also recently been applied to proteomics and metabolomics data analysis (DiLeo et al., 2011). WGCNA can be used to identify candidate biomarkers or therapeutic targets, and has been used in a variety of human cancers, including colon cancer, uveal melanoma, glioblastoma, liver cancer, and osteosarcoma (Langfelder and Horvath, 2008). This study saw a focus on the gene co-expression network of annulus fibrosus and the principal cause of discogenic symptoms (Kazezian et al., 2015). Integrated bioinformatics methods, including WGCNA, were applied to generate a comprehensive overview of the gene network associated with IDD. Expression of some of the identified hub genes was verified using clinical samples. These hub genes might represent novel therapeutic targets for IDD.

## Materials and Methods

### Date Acquisition and Clinical Samples

Under the accession number GSE70362, the download of the data series was accessed from Gene Expression Omnibus (www.ncbi.nlm.nih.gov/geo/). Processed data of annulus fibrous were selected for this study. The filtration of DEGs of degenerative (grade III–V) vs. non-degenerative (grade I–II) intervertebral disc samples was achieved using a two-tailed *t*-test. Using DEGs for further analysis not only preserved variance in genetic background, but also reduced unrelated genetic noise. We collected the specific clinical characteristics of clinical samples from 10 patients with IDD, including gender, age, level, and pfirrmann grade.

### Patients and Tissues

Ethics Review Board of Renji Hospital (number 2017-003) approved the study trials and the study was performed in accordance with the rules of the China Food and Drug Administration/Good Clinical Practice and the Declaration of Helsinki (2008) of the World Medical Association. All participants or their parents/legal guardians for patients aged under 18 years provided the written informed consent.

From patients with IDD in the Spine Group of Renji Hospital, degenerative intervertebral disc tissue samples were obtained. Patients with IDD combined with infections, tumors, or previous lumbar disc surgery were not included in this study. From patients with accidental fractures, non-degenerative specimens were collected. None of the patients in the non-degenerative group reported any previous lumbar pain. Based on the Pfirrmann grading system, the degenerative condition was evaluated by two independent observers using magnetic resonance imaging (Pfirrmann et al., 2001). All the intervertebral disc specimens were collected within 1 h after disc excision, rinsed with phosphate-buffered saline and then stored in the RNAstore Reagent DP408-02 (Tiangen Biotech, Beijing, China) at 4°C.

### Gene Ontology Analysis and Pathway Analysis

The application of gene ontology (GO) analysis to upregulated and downregulated genes were operated separately (Ashburner et al., 2000). According to Gene Ontology Consortium, GO classifies gene functioned in a species-independent way in line with three aspects: cellular component, molecular function and biological process. GO analysis was performed to determine the GO terms that were over- or under-represented in a given set of genes. GO analysis was performed using ClueGO to generate a visual representation of the enriched terms in a functionally grouped annotation network which reflected the relationships between enriched terms. The leading term in a group was the most significant (Bindea et al., 2009). The *p*-value of enrichment analysis should be <0.05.

By interrogating the Kyoto Encyclopedia of Genes and Genomes (KEGG) database, the Database for Annotation, Visualization and Integrated Discovery (DAVID, http://david.abcc.ncifcrf.gov/) was used to achieve pathway analysis (Huang et al., 2009; Kanehisa et al., 2017). The *p*-value of enrichment analysis should be <0.05. The Ingenuity Pathway Analysis Database (IPA, www.Ingenuity.com) was also used for pathway analysis. KEGG pathway analysis is a topology-based approach which takes into account gene interactions whereas IPA is based on gene expression (Khatri et al., 2012). We used a combination of KEGG pathway analysis and IPA to generate more complete and accurate information about the identified DEGs. Up- and down-regulated genes were subjected to KEGG pathway analysis separately. The fold changes in gene expression of the up- and down-regulated genes subjected to IPA. The *p*-value of enrichment analysis should be <0.05.

### Weighted Gene Co-expression Network Analysis

Through employing static programming language and environment R, Weighted Gene Co-expression Network Analysis was conducted with WGCNA package (Jiang et al., 2014; Core R, 2015). Only DEGs were included in the WGCNA workflow to minimize noise and reduce the computing burden without causing major information loss (Ghazalpour et al., 2006). The adjacency matrix was calculated based on pairwise Pearson correlation coefficients. WGCNA incorporated the concept that genes interactions occurred following a scale-free distribution pattern (Barabasi, 2009). The *pickSoftThreshold* function was applied to fit the scale-free criterion. Topologic overlap measures, which were a robust measure of networks, were calculated pairwise within the adjacency matrix. The dynamic tree cutting algorithm, an unsupervised hierarchical clustering method, was adopted for clustering with input of topologic overlap measures (Langfelder et al., 2008). In this study, the soft threshold (power) was 4. We used the default parameters in WGCNA algorithm, the maximum size of module was 500, and the minimum size was 30.

Modules can be explained as branches of the clustering tree. In network terminology, a module refers to a group of genes that share similar connection patterns with all other genes outside that group and there are, generally speaking, similar functions existing in genes in the same module (Zhang and Horvath, 2005). The calculation of the main component of module, a module eigengene, was then conducted to summarize the gene expression profiles in the module. In order to identify the modules that were most related to IDD for further analysis, the calculation of correlations between module eigengenes and the degenerative status of samples was operated.

In a scale-free network, hub genes of modules are the most interconnected genes and they serve as the backbones of this network (de Jong et al., 2012). Hub genes in disease-related modules, such as hub lncRNA in IDD, are generally biologically and clinically meaningful (Jiang et al., 2014; Lee et al., 2014; Chen et al., 2015; Wang et al., 2015). Hub genes were determined through ranking intra-modular connectivity and correlation with eigengenes in selected module. Gene co-expression networks of all DEGs and hub genes were visualized using Cytoscape (Shannon et al., 2003).

### Ingenuity Pathway Analysis of Selected Modules and Hub Genes

Genes in selected modules were subjected IPA to evaluate their relationship with IDD. GO analysis and KEGG pathway analysis were commonly performed. However, these types of analysis considered only the number of genes in a given set and ignored any values related to genes (Khatri et al., 2012). We undertook a close examination of selected modules including both up- and down-regulated genes. Thus, GO and KEGG analyses of heterogeneous data such as ours were inappropriate. Instead, we performed IPA, which also took into account gene expression levels. This workflow has been widely adopted in many other weighted gene co-expression network analyses (Naylor et al., 2010; Malki et al., 2013). We also adopted the Disease and Biofunction module of IPA which was similar to Go Analysis.

### Validation of Hub Gene Expression

To validate the expression pattern of some hub genes, quantitative real-time PCR (qRT-PCR) was conducted. TRIzol reagent (Invitrogen, Carlsbad, CA, USA) was employed to extract RNA based on the instructions of the manufacturer, and qRT-PCR assays were conducted through adopting the ViiA7 Real-Time PCR System (Applied Biosystems, CA, USA) with a thermal profile comprising one min at 95°C for polymerase activation, followed by 40 cycles of 95°C for 15 s and 60°C for 30 s. Expression of target genes was normalized to ß-actin as the endogenous control. For statistical analysis, the calculation of gene expression was processed following the 2^−ΔΔCt^ method, and relative expression data were log2 transformed (Livak and Schmittgen, 2002). The list of sequences of primers used for qRT-PCR amplification was presented in Supplementary Table 1.

### Statistical Analysis

All quantitative data were represented as mean ± SD. In the mRNA expression experiments (SPSS Statistics Version 22.0; IBM Corp, Armonk, NY, USA), in order to compare control groups with the IDD group, student's *t*-test was operated. Unless otherwise stated, when *P*-values were below 0.05, differences were taken as statistically significant.

## Results

### Clinical Characteristics of Samples

The specific clinical traits of all samples in GSE70362 were provided in Supplementary Table 2. From levels T12–L1 to L4–L5, five pairs of non-degenerative and degenerative annulus fibrous samples were collected for qRT-PCR analysis to confirm hub gene expression. Specific clinical traits of these sample were provided in Supplementary Table 3. In the degenerative group, the average age was 48.0 years (range, 33–61 years) with Pfirrmann Grade III–V disc. In the non-degenerative group, the average age was 31.8 years (range, 16–52 years) with Pfirrmann Grade I–II disc.

### Gene Ontology Analysis and Pathway Analysis

Altogether 2,636 probes were identified as differentially expressed in comparisons of degenerative and non-degenerative annulus fibrosus tissue samples. According to the annotation file, 1,190 probes were mapped to known genes (464 upregulated and 726 downregulated).

**Figure 1:**
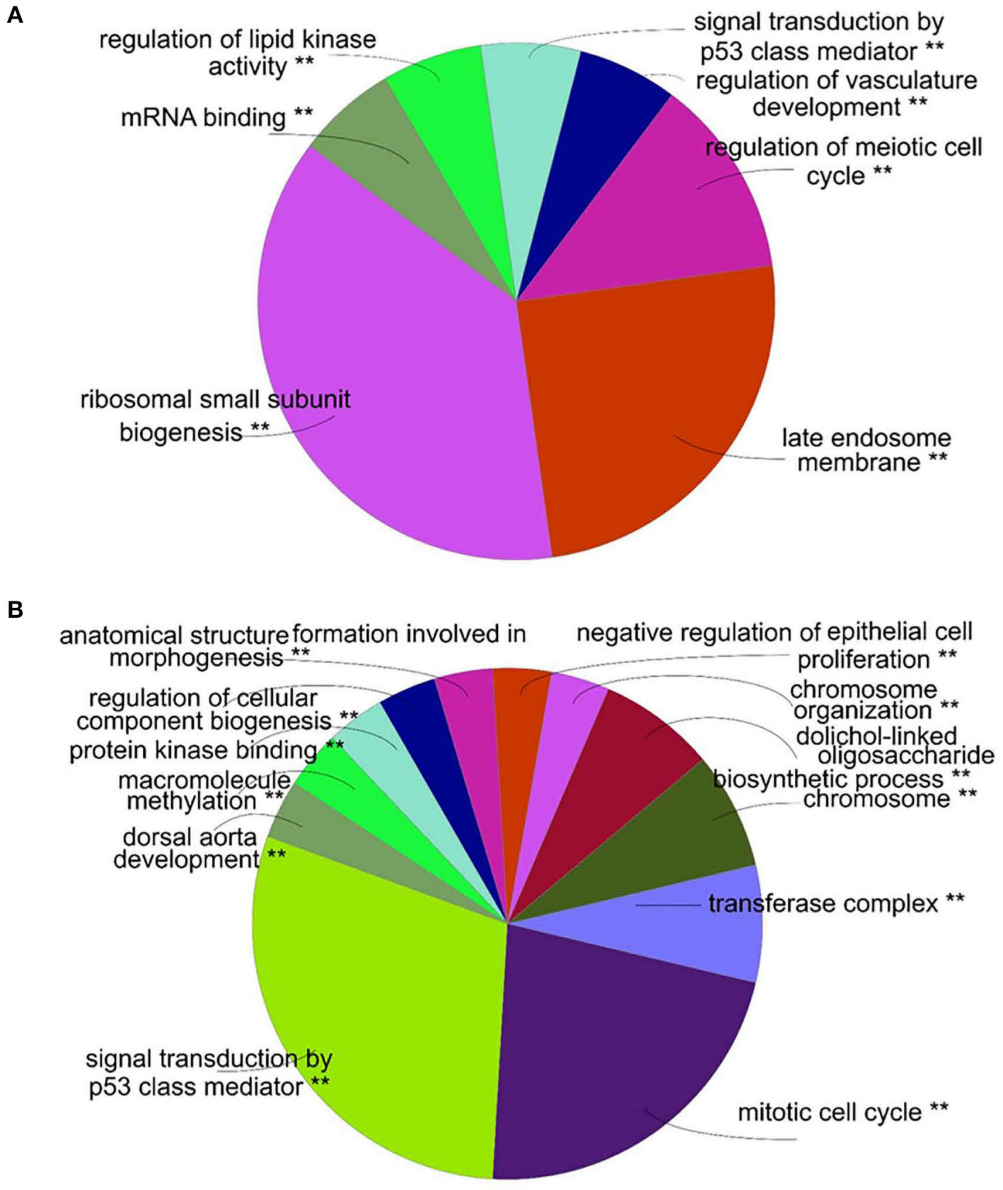
Gene ontology (GO) analysis of differentially expressed genes. **(A)**. GO analysis of upregulated genes by ClueGO. According to the most significant gene in the group, each section of the overview pie-chart represents a group of GO terms with section names allocated. The correlation exists between the size of each section and the number of genes within the group. **(B)**. GO analysis of downregulated genes by ClueGO. **P < 0.01.

An overview of the GO analysis was presented in Figure 1 and specific results for up- and downregulated genes are provided in Supplementary Figures 1, 2, respectively. For both up- and down-regulated genes, signal transduction by a p53 class mediator was enriched, indicating the involvement of apoptosis in IDD. For upregulated genes, regulation of vasculature development was enriched, which was consistent with the vascularization associated with IDD (Freemont et al., 1997).

Results of pathway analysis were presented in Table 1. A long list of activated pathways has been generated by IPA and only the IDD-related pathways were presented. The complete IPA results were presented in Supplementary Table 4. The TNF signaling pathway, which had a well-established association with IDD, was found to be activated in KEGG pathway analysis of upregulated genes and TNFR1 and TNFR2 signaling activation was identified by IPA (Risbud and Shapiro, 2014). TGF-β signaling was also identified by IPA. Other cytokine signaling pathways including B cell activating factor signaling, IL-1 signaling and IL-6 were also identified (Supplementary Table 4) confirming the role of inflammation in IDD. Mismatch repair signaling was identified in both KEGG pathway analysis and IPA. Furthermore, apoptosis signaling activation was identified, thus confirming the results of GO analysis. Axonal guidance signaling was also identified, which highlighted the role of neural ingrowth in IDD (Freemont et al., 1997; Kepler et al., 2013).

**Table 1:** Pathway analysis of differentially expressed genes.

**Term**	***P*-value**
**KEEG UP-REGULATED GENES**	
Progesterone-mediated oocyte maturation	0.00561
HTLV-I infection	0.00631
TNF signaling pathway	0.012669
Pathways in cancer	0.013666
Regulation of actin cytoskeleton	0.020803
ErbB signaling pathway	0.027356
Prostate cancer	0.028379
Mismatch repair	0.036276
Acute myeloid leukemia	0.037105
Hepatitis B	0.041927
Choline metabolism in cancer	0.043744
Colorectal cancer	0.047879
**KEEG DOWN-REGULATED GENES**	
DNA replication	0.034
Cell cycle	0.036
**IPA**	
TNFR2 signaling	2.75E-05
Mismatch repair in eukaryotes	8.91E-05
Induction of apoptosis by HIV1	9.77E-05
Apoptosis signaling	0.001995
B cell activating factor signaling	0.008913
Axonal guidance signaling	0.009333
HGF signaling	0.01349
Acute phase response signaling	0.016218
TNFR1 signaling	0.017783
TGF-β signaling	0.022387

*The analysis of up- and downregulated genes by Kyoto Encyclopedia of Genes and Genomes (KEGG) is shown separately (only pathways with P <0.05 are shown). Ingenuity pathway analysis (IPA) analysis results are customized to display related pathways only; complete results are shown in Supplementary Table 4*.

### Weighted Gene Co-expression Network Analysis

**Figure 2:**
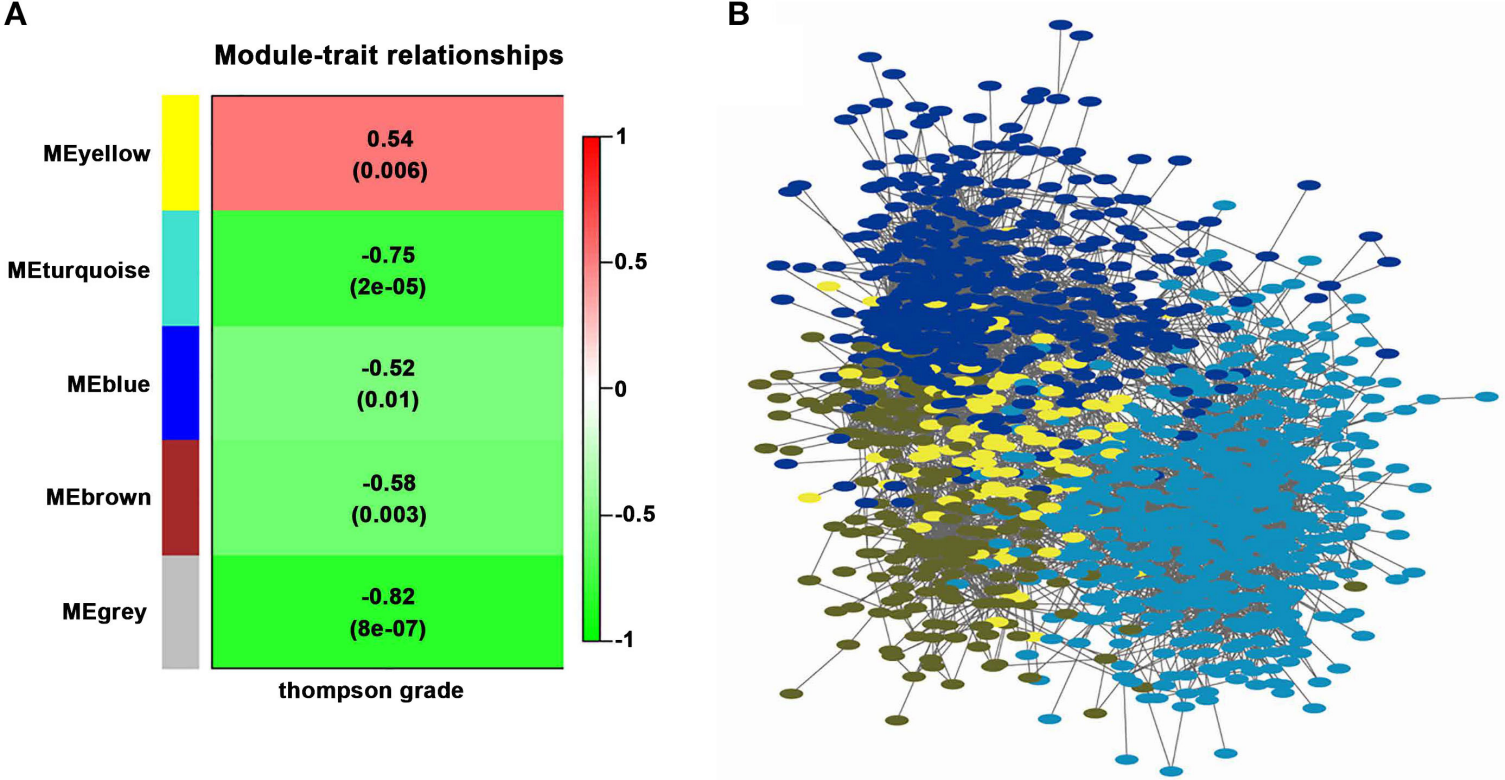
Weighted gene co-expression network of annulus fibrosus in IDD. **(A)**. Correlation between module and Thompson Grade. Each cell represents a module with the corresponding correlation (above) and *P*-value (below). The names of the modules are presented to the left of cells and the bar on the right represents correlation values. **(B)**. Visual representation of Weighted Gene Co-expression Network. The node represents differentially expressed gene, the edge represents the interaction between two genes.

Four modules were generated by WGCNA; these modules were identified by different colors and genes that could not been classified into any modules were shown in gray. In WGCNA's algorithm, some non-clustering genes will be put into a single module, which will be uniformly called “gray.” Correlation between modules and Thompson Grade were shown in Figure 2A. The module with the lowest *P*-value (shown in turquoise) and the highest absolute correlation coefficient was considered to be the module which is most related to IDD and selected for further analysis. A visual representation of the whole weighted gene co-expression network was shown in Figure 2B. Nodes represent genes and node color indicated module membership. Correlation existed between edges between nodes and topologic overlaps (analogous to correlation), genes and small distances indicate high correlation. There was a tendency for genes within the same module to stay close to each other in the weighted gene co-expression network by visual inspection of Figure 2B. The complete results of WGCNA were provided in Supplementary Table 5.

The gene is represented by each node while the module membership is indicated by node color. Correlation exists between edges between nodes and topologic overlaps (analogous to correlation) between genes and small distances indicate high correlation. The purpose of this research was to find hub module, and the turquoise module was highly correlated with the disease. Therefore, we focused on the analysis of this module.

**Figure 3:**
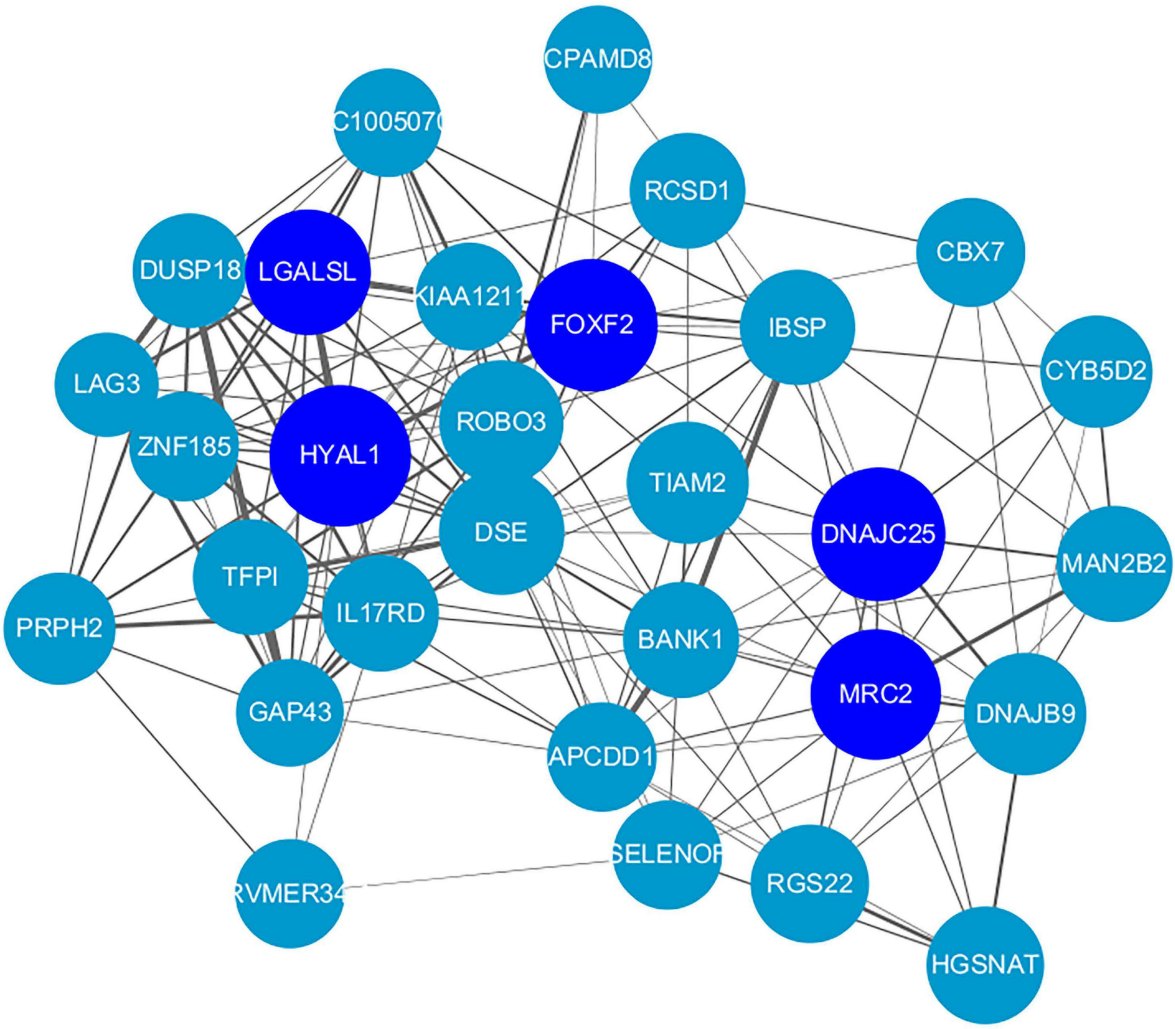
Top 30 hub genes in the turquoise module. Hub genes are represented by nodes and correlation exists between node size and the intra-modular connectivity of the gene. In the center of this network, the top 10 hub genes are located and they are shown in darker colors.

By ranking intra-modular connectivity and correlation with the module eigengene, hub genes in the turquoise module were identified. The top hub genes in the turquoise module were represented in Figure 3. To be clarified, only the top 30 hub genes were included. Hub genes were represented by nodes and correlation exists between node size and the intra-modular connectivity of the gene. The selection criteria of hub genes was the top 10 genes with the highest connectivity in the co-expression network. DSE, IL17RD, DUSP18, ROBO3, BANK1, MRC2, LGALSL, TFPI, GAP43, and HYAL1, the top ten hub genes, were shown in darker colors.

### Ingenuity Pathway Analysis of Turquoise Module

**Figure 4:**
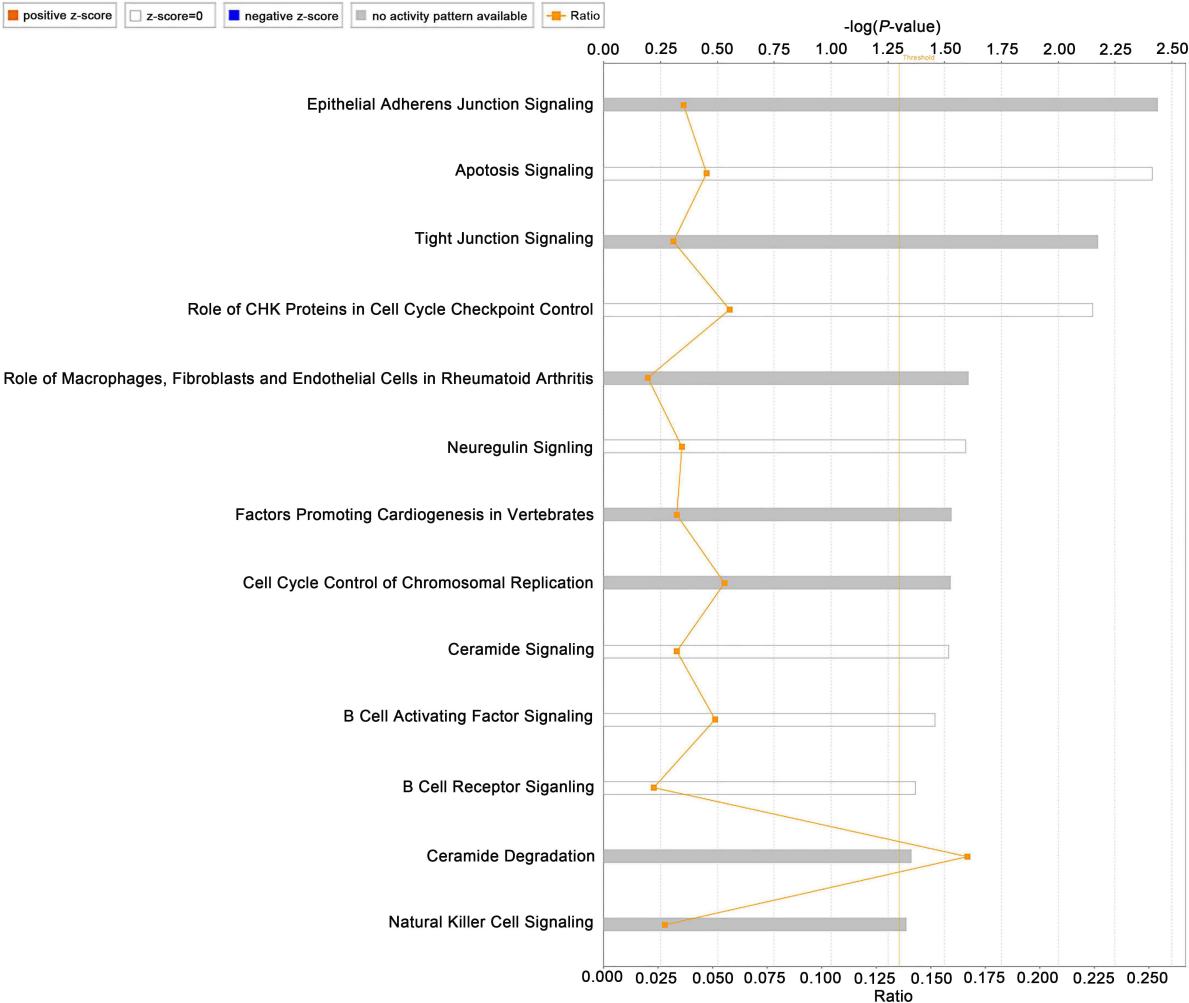
Ingenuity pathway analysis of the turquoise module. Left part shows pathway names and bars represent the corresponding pathway's –log (*P*-value).

To evaluate the relationship between the turquoise module and IDD, IPA was performed. As shown in Figure 4, “apoptosis signaling,” “factors promoting cardiogenesis invertebrates,” “neuregulin signaling,” “B cell receptor signaling” “B cell activating factor signaling” and “natural killer cell signaling” were identified by IPA. These pathways highlighted the role of apoptosis, neural ingrowth, vascularization, and inflammatory cytokines in IDD. Highly related pathways were also identified in the Diseases and Bio Functions module (Supplementary Figure 3).

### Confirmation of Hub Gene Expression

**Figure 5:**
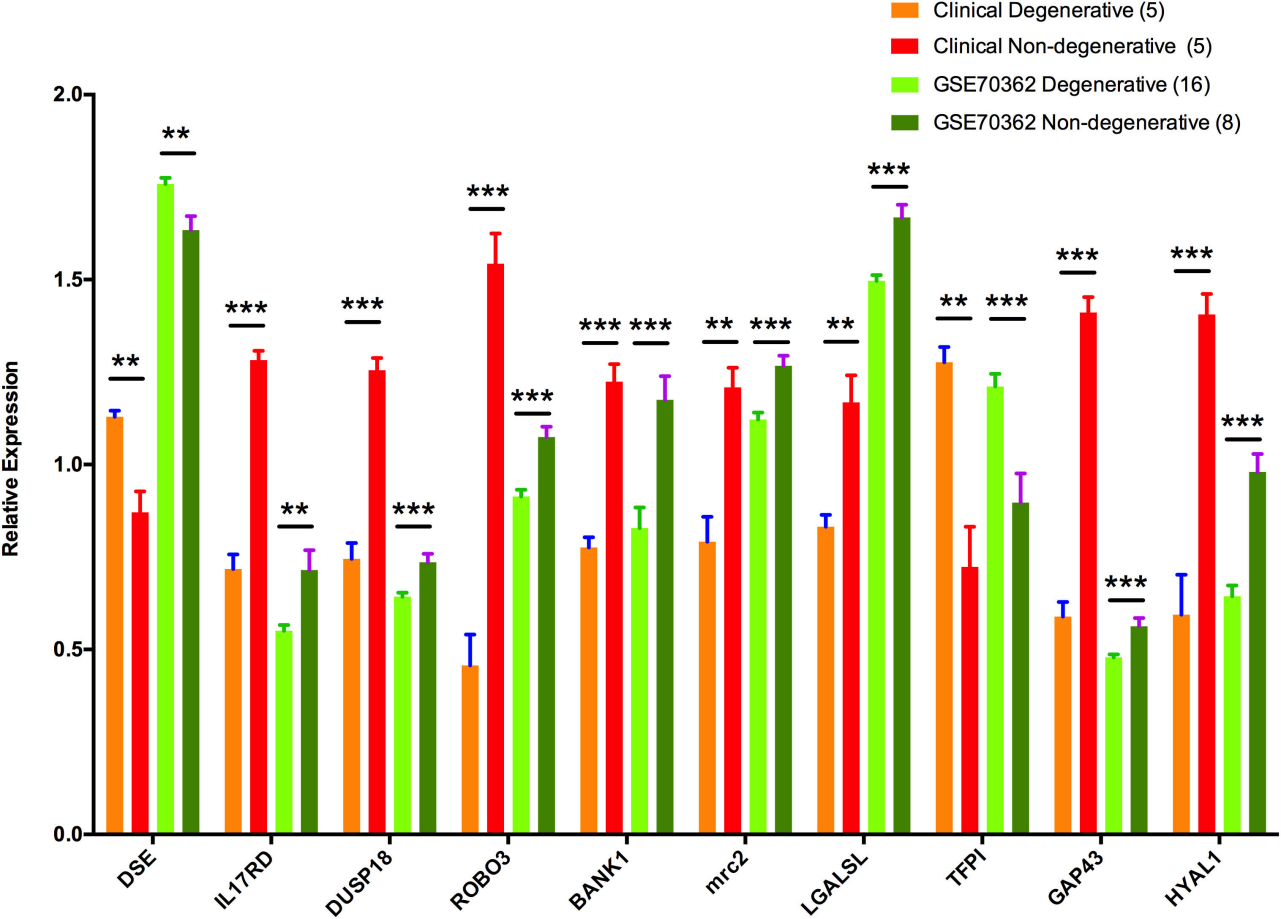
Relative expression levels of 10 hub genes. Expressions of genes in GSE70362 and clinical samples. ^**^0.001 < *P* < 0.01; and ^***^P < 0.001.

To validate the outcomes of the turquoise module analysis and to identify key genes involved in IDD, RT-PCR was employed to analyze the relative expression levels of 10 hub genes. Comparisons of degenerative disc and matched non-degenerative disc samples revealed significant downregulation of eight genes (IL17RD, DUSP18, GAP43, and HYAL1 [*P* < 0.001]; ROBO3, BANK1, and MRC2 [*P* < 0.01]; LGALSL [*P* < 0.05]) and significant upregulation of two genes (TFPI [*P* < 0.001]; DSE [*P* < 0.01]). This expression profile was consistent with the microarray data (Figure 5). To make data from PCR and microarray comparable, expression values of each hub genes were transformed by dividing the average value in whole expression cohort from corresponding data source.

## Discussion

This study applied integrated bioinformatics approaches to identify variations in gene expression related to annulus fibrous in degenerative and non-degenerative intervertebral disc tissues. We generated a complete overview of the gene networks to highlight gene modules and hub genes highly related to IDD. The biological and clinical importance of hub genes in weighted gene co-expression networks have been widely reported (Chen et al., 2015). This study identifies the hub genes which may be of importance to the pathogenesis of IDD. By applying this novel method of analysis, the present study not only updates our perspective on the pathogenesis of IDD, but also highlights some hub genes which have the potential to be IDD biomarkers and treatment targets.

GO and pathway analyses revealed differences in annulus fibrous associated with degenerative and non-degenerative intervertebral disc tissue. Some apoptosis, neural ingrowth, vascularization, and inflammation related terms and pathways were identified that were consistent with well-established molecular mechanisms of IDD (Kepler et al., 2013). The pathogenesis of IDD includes cellular oxidative stress, mitochondrial dysfunction and apoptosis (Kang et al., 2019). Endplate chondrocyte apoptosis is an important cause of the pathogenesis of cartilage endplate (CEP) degeneration, leading to the occurrence and development of intervertebral disc degeneration (IDD) (Wu et al., 2010; Li et al., 2014). Nucleus pulposus (NPC) apoptosis is the main factor of IDD. Nucleus pulposus (NP) cell apoptosis is a classic cell characteristic in the IDD process (Xianzhou and Cunxin, 2018). The vascularization of the intervertebral disc is generally considered to be a pathological feature of IDD (Johnson et al., 2007). As IDD progresses, intervertebral disc tend to be increasingly vascularized through angiogenesis. Recent evidence suggests that in addition to abnormal and excessive mechanical loads, inflammation may be a key driver of IDD and low back pain (Sharma, 2018). A study by GSE70362 has identified various dysfunctional cell functions, including cell proliferation and inflammation, and similar findings have been found in this study (Kazezian et al., 2015). Human T lymphovirus type I (HTLV-I) is the inducer of adult T-cell leukemia/lymphoma and HTLV-1-related myelopathy (Sherman et al., 1993). HTLV-I can cause chronic infections that cannot be cured or neutralized by vaccines. Due to HTLV-1 infection, the overall risk of death increases. The research of GSE70362 found that the most important classical pathway induced in degeneration fibrosus was the interferon pathway (Kazezian et al., 2015). Other famous pathways including TNF and TGF-β signaling were also determined in this study (Freemont, 2009). It is well known that the tumor necrosis factor TNF pathway affects the survival of cancer patients (Yi et al., 2018). TNF signal responds to cellular stress and inflammation signals, activates pro-apoptotic pathways and cytokine cascades (Chau et al., 2005). Transforming growth factor-beta (TGF-β) is a cytokine necessary to induce fibrosis and activate cancer stroma (Busch et al., 2015; Chen et al., 2019). The TGF-β signaling pathway plays an important role in many biological processes, including cell growth, differentiation, apoptosis, migration, and the occurrence and development of cancer (Waddell et al., 2004). Four gene modules were generated by WGCNA, and among them, the module that was most highly related to IDD was the turquoise module. Further analysis by IPA validated its tight correlation with IDD. By ranking intra-modular connectivity and correlation with the module eigengene, hub genes in the turquoise module were identified. Using this approach, DSE, IL17RD, DUSP18, ROBO3, BANK1, MRC2, LGALSL, TFPI, GAP43, and HYAL1 were identified as the top 10 hub genes. Hub gene expression profiles were confirmed by RT-PCR analysis.

Hub genes such as DSE, MRC2, and HYAL1 have a considerable effect on extracellular matrix metabolism, alterations in which are a major cause of IDD (Le Maitre et al., 2007). The DSE gene encodes dermatan sulfate epimerase, which regulates the biosynthesis of dermatan sulfate, an important element of the extracellular matrix. Furthermore, DSE-deficient mice have altered collagen structure (Maccarana et al., 2009). MRC2 is a versatile mediator of extracellular matrix metabolism and regulates not only collagen internalization, but also matrix metalloproteinase activity (Bailey et al., 2002; Messaritou et al., 2009; Madsen et al., 2011; Jurgensen et al., 2014). MRC2 also regulates TGF-β function (Caley et al., 2012). The HYAL1 gene encodes lysosomal hyaluronidase, which catalyzes the degradation of hyaluronan, which is one of the major glycosaminoglycans of the extracellular matrix (Lokeshwar et al., 2006). In addition, HYAL1 degenerates chondroitin sulfate, which is also an important component of extracellular matrix (Gushulak et al., 2012).

Neural ingrowth is reported to be involved in the pathogenesis of IDD, and our analysis indicates the involvement of hub genes ROBO3 and GAP43 in this process (Freemont, 2009; Kepler et al., 2013). ROBO3 is proposed to be involved in guiding neuronal axon growth, while GAP43 plays a well-established role in neuronal development and plasticity (Serin et al., 2016).

Inflammation is an essential participant in IDD and both IL17RD and BANK1 are important mediators of inflammatory reactions (Risbud and Shapiro, 2014; Molinos et al., 2015). IL17RD, which interacts with the IL-17 receptor to initiate IL-17 signaling, has been proposed as a therapeutic target in axial spondyloarthritis (Rong et al., 2009; Paine and Ritchlin, 2016). BANK1 mediates B cell signaling is involved in autoimmune disease such as systemic lupus erythematosus (Bernal-Quirós et al., 2013).

The hub gene TFPI may be a versatile participant in IDD based on its ability not only to regulate angiogenesis, but also to induce apoptosis (Hamuro et al., 1998; Amirkhosravi et al., 2007; Fu et al., 2008). Although these hub genes are well-characterized, the remaining two hub genes, DUSP18 and LGALSL, have not been researched extensively. Thus, the potential mechanisms by which these genes participate in the pathogenesis of IDD remain to be clarified. Nevertheless, the close relationships of the other eight hub genes with IDD indicates an important role for DUSP18 and LGALSL.

The major limitation of the present study is the isolated analysis of annulus fibrosus data. Although the formation of vascularized granulation tissue and innervation in annulus fibrosus are the principal causes of discogenic symptoms, the role of nucleus pulposus cannot be ignored (Livak and Schmittgen, 2002). Therefore, the integrated bioinformatics approaches adopted in this study will be used to explore how nucleus pulposus functions in IDD. This combined analysis of annulus fibrosus and nucleus pulposus data will provide a more integrated overview of the gene networks involved in IDD.

In conclusion, the present study was conducted using integrated bioinformatics approaches to generate a comprehensive overview of the gene network associated with annulus fibrosus in IDD. We identified 10 hub genes, DSE, IL17RD, DUSP18, ROBO3, BANK1, MRC2, LGALSL, TFPI, GAP43 and HYAL1, which updated our perspective on the pathogenesis of IDD, and could also serve as novel biomarkers and potential therapeutic targets. In addition, we also explore related signal transduction pathways and interaction networks. IDD is the main contributor to low back pain, which is the main cause of disability worldwide. This study provides clues to the molecular mechanism of IDD for future experimental studies. At the same time, this shows that bioinformatics methods can be used to identify potential targets for other human tumors.

## Data Availability Statement

The datasets presented in this study can be found in online repositories. The names of the repository/repositories and accession number(s) can be found in the article/Supplementary Material.

## Ethics Statement

Written informed consent was obtained from the individual(s) for the publication of any potentially identifiable images or data included in this article.

## Author Contributions

BC and HW: conception and design. HW and WL: development of methodology. XY: sample collection. HW, BY, and WL: analysis and interpretation of data. HW, WL, and BC: writing, review, and/or revision of the manuscript. All authors contributed to the article and approved the submitted version.

## Conflict of Interest

The authors declare that the research was conducted in the absence of any commercial or financial relationships that could be construed as a potential conflict of interest.
