# Supplementary figures and images for: Identification of Key Modules and Hub Genes of Annulus Fibrosus in Intervertebral Disc Degeneration
Source: Front Genet. 2021 Jan 27;11:596174. doi: 10.3389/fgene.2020.596174 (PMC7875098; doi:10.3389/fgene.2020.596174)

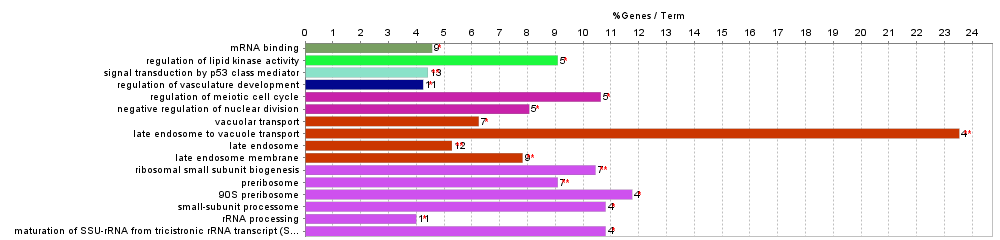

Supplement: Supplementary file 6 [file Image_1.TIFF]

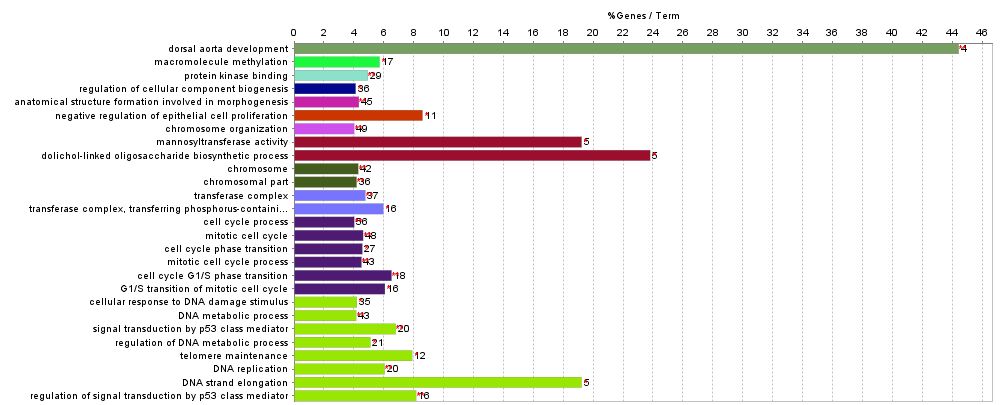

Supplement: Supplementary file 7 [file Image_2.TIFF]

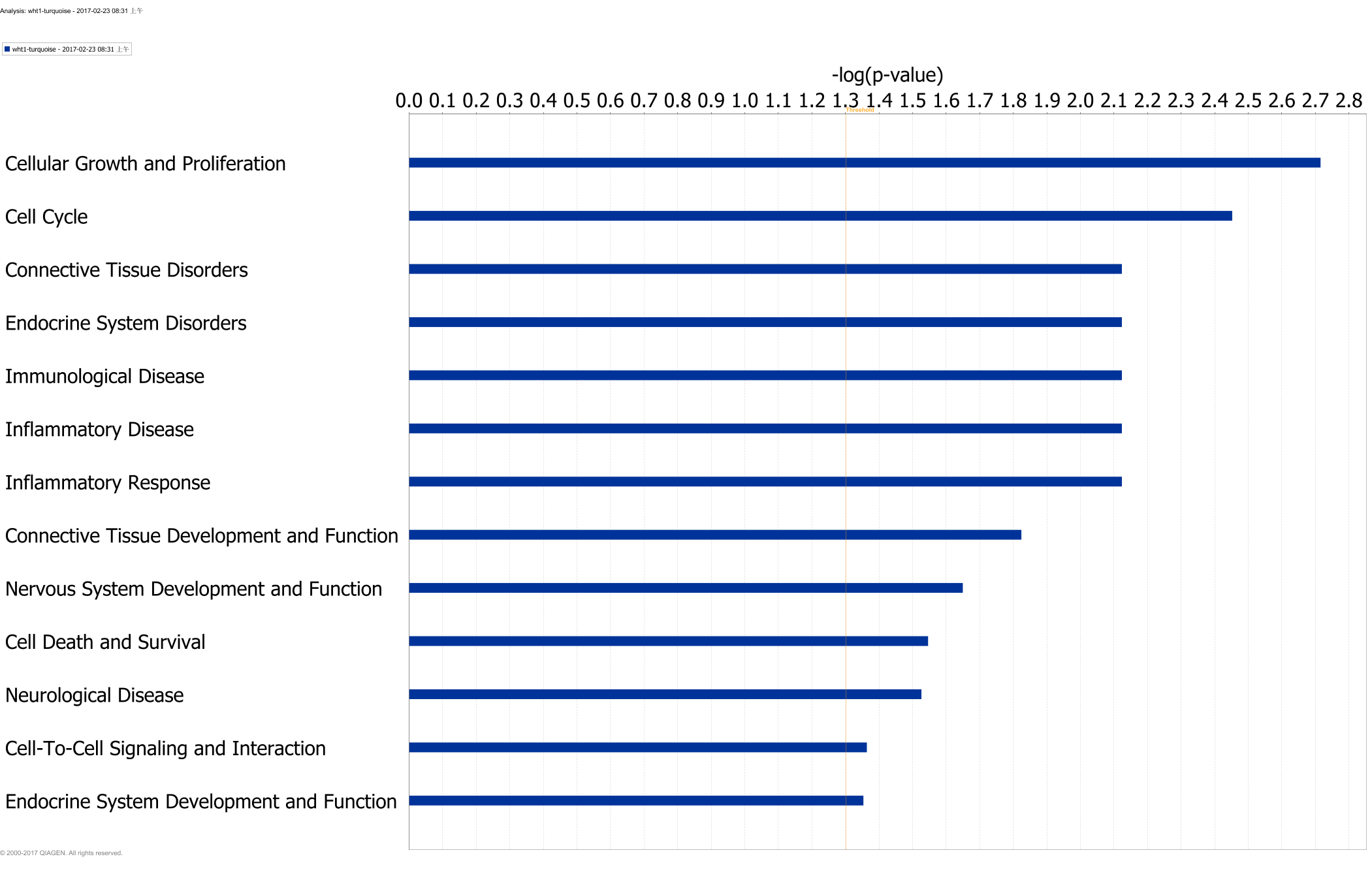

Supplement: Supplementary file 8 [file Image_3.TIFF]
